# Site‐Selective Biofunctionalization of 3D Microstructures Via Direct Ink Writing

**DOI:** 10.1002/smll.202404429

**Published:** 2024-09-18

**Authors:** George Mathew, Enrico Domenico Lemma, Dalila Fontana, Chunting Zhong, Alberto Rainer, Sylwia Sekula‐Neuner, Jasmin Aghassi‐Hagmann, Michael Hirtz, Eider Berganza

**Affiliations:** ^1^ Institute of Nanotechnology (INT) Karlsruhe Institute of Technology (KIT) Kaiserstraße 12 76131 Karlsruhe Germany; ^2^ Karlsruhe Nano Micro Facility (KNMFi) Karlsruhe Institute of Technology (KIT) Kaiserstraße 12 76131 Karlsruhe Germany; ^3^ Department of Engineering Università Campus Bio‐Medico of Rome via Álvaro del Portillo 21 Rome 00128 Italy; ^4^ Fondazione Policlinico Universitario Campus Bio‐Medico di Roma via Álvaro del Portillo 200 Rome 00128 Italy; ^5^ Institute of Nanotechnology (NANOTEC) National Research Council via Monteroni Lecce 73100 Italy; ^6^ n.able GmbH Hermann‐von‐Helmholtz‐Platz 1 76341 Eggenstein‐Leopoldshafen Germany; ^7^ Instituto de Ciencia de Materiales de Madrid (CSIC) c) Sor Juana Inés de la Cruz, 3 Madrid 28049 Spain

**Keywords:** 3D cell culture, dip‐pen nanolithography, direct laser writing, phospholipids, proteins, surface functionalization, two‐photon lithography

## Abstract

Two‐photon lithography has revolutionized multi‐photon 3D laser printing, enabling precise fabrication of micro‐ and nanoscale structures. Despite many advancements, challenges still persist, particularly in biofunctionalization of 3D microstructures. This study introduces a novel approach combining two‐photon lithography with scanning probe lithography for post‐functionalization of 3D microstructures overcoming limitations in achieving spatially controlled biomolecule distribution. The method utilizes a diverse range of biomolecule inks, including phospholipids, and two different proteins, introducing high spatial resolution and distinct functionalization on separate areas of the same microstructure. The surfaces of 3D microstructures are treated using bovine serum albumin and/or 3‐(Glycidyloxypropyl)trimethoxysilane (GPTMS) to enhance ink retention. The study further demonstrates different strategies to create binding sites for cells by integrating different biomolecules, showcasing the potential for customized 3D cell microenvironments. Specific cell adhesion onto functionalized 3D microscaffolds is demonstrated, which paves the way for diverse applications in tissue engineering, biointerfacing with electronic devices and biomimetic modeling.

## Graphical abstract

A combination of two‐photon lithography with scanning probe lithography is proposed as a new approach for biofunctionalization of 3D microstructures, with spatially controlled biomolecule distribution. The method utilizes a diverse range of biomolecule inks, including phospholipids, and two different proteins, introducing sub‐1 µm resolution and distinct functionalization on separate areas of the same microstructure, for different types of photoresists.

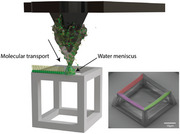

## 1 Introduction

The emergence of two‐photon lithography (2PL) has transformed the landscape of multi‐photon laser printing, ushering in a new era for the production of 3D micro‐ and nanoscale structures.^[^ ^1^ ^]^ This direct laser writing (DLW) technique employs a tightly focused femtosecond laser in the near‐infrared spectral range, triggering nonlinear multiphoton absorption processes that facilitates the formation of 3D microarchitectures by manipulating the focal point position through a suitable photocurable ink.^[^ ^2^, ^3^ ^]^ The resolution of 2PL, writing speed and capability to fabricate on demand 3D microstructures has boosted advances in many fields and applications, from microfluidics^[^ ^4^ ^]^ to microrobotics,^[^ ^5^ ^]^ metamaterials,^[^ ^6^ ^]^ or tissue engineering.^[^ ^7^ ^]^ However, 2PL encounters limitations due to the restricted availability of functional resist formulations, which constitutes one of the major bottlenecks toward the creation of functional or stimuli responsive microstructures. Rapid expansion of this field has triggered research aimed at developing different strategies to create microscaffolds with properties that vary when subjected to external physical and chemical cues (4D printing).^[^ ^8^ ^]^ The most extensively studied stimuli include temperature, light, pH, and magnetic and electric fields.

Recent studies have introduced innovative photoresists for multiphoton polymerization (MPP) and stimulated emission depletion (STED) lithography, resulting in stable nanostructures with reactive groups for covalent modifications. Combining 3D printing with advanced surface modifications enhances functionality, creating surfaces with unique properties for biomedicine, engineering, and electronics. Techniques like laser‐Assisted protein adsorption by photobleaching (LAPAP) and trichlorovinylsilane (TCVS) treatment with thiol–ene chemistry create highly functionalized scaffolds and surfaces. Surface radical polymerization provides precise control of surface chemistry, while MPP and (DLW) with post‐fabrication modifications enable complex devices and biomimetic cell culture environments, expanding the potential of 3D‐printed materials and biomaterials.^[^ ^9^, ^10^, ^11^, ^12^, ^13^, ^14^ ^]^

One of the most common approaches aiming at functionalization relies on making changes to the formulation of the photoresist, usually achieved by adding stimuli‐responsive monomers that contain acrylates and functional groups.^[^ ^15^ ^]^ An alternative approach consists on the utilization of nanofillers in nanocomposites,^[^ ^16^ ^]^ which despite endowing functionality, present considerable drawbacks, such as the limited load of nanofillers achievable, as the photoresist needs to remain transparent to the laser light. Additionally, the presence of nanoparticles or other nanoelements may compromise the resolution and stability compared to a single‐phase photoresist blend, due to laser scattering in the presence of small particles while printing. Finally, the use of post‐functionalization strategies, such as the chemical modification of a microstructure surface^[^ ^17^ ^]^ or the growth of a thin film to render the microstructure magnetic‐field responsive can be often found in some fields.^[^ ^18^, ^19^ ^]^

In the domain of biofunctionalization, prior efforts have predominantly focused on employing commercially available photoresists for applications in cell biology and nanoelectronics.^[^ ^20^ ^]^ However, many studies utilizing single photoresist scaffolds with a homogeneous surface coating of biomolecules face challenges in achieving spatially controlled distribution of biomolecules. Given that biomolecules are typically unevenly distributed in tissues, a patterned distribution of proteins in 3D microstructures becomes essential. Initial attempts toward site‐selective biofunctionalization involved a two‐component scaffold,^[^ ^21^ ^]^ allowing controlled cell attachment but limited to a single type of protein.^[^ ^22^ ^]^ Advancements included introducing chemical groups for dual biomolecule coating, including DNA, via light‐induced click reactions, which requires processing with focused UV laser sources.^[^ ^23^ ^]^ Alternative strategies require the use of specific protein linkers, posing challenges in expression and purification. Using proteins as 2PL resists for multifunctional scaffolds is an intriguing option, yet questions persist about their functionality and mechanical stability.^[^ ^24^ ^]^ These challenges highlight the ongoing need for innovative biofunctionalization approaches, which are site‐specific, include multiplexing capabilities and endow high versatility for the introduction of custom biomolecules.

Addressing this challenge, our novel approach proposes a post‐functionalization strategy,^[^ ^25^ ^]^ combining the use of the 2PL to build a microscaffold, with scanning probe lithography (SPL) techniques,^[^ ^26^ ^]^ such as Dip‐Pen Nanolithography (DPN) and Microchannel Cantilever Spotting (µCS), for site‐selective biofunctionalization. This synergistic use of techniques not only allows for the integration of a wide range of different biomolecules on the surface of 3D microscaffolds with high spatial resolution, but also enables distinct functionalization on different areas of the same microscaffold: multiplexing. For simplicity, proof of concept and many optimization experiments have been conducted using a 1,2‐dioleoyl‐sn‐glycero‐3‐phosphocholine (DOPC),^[^ ^27^, ^28^ ^]^ a fluid phospholipid ink as model ink, before moving on to the different examples for other classes of biomolecules. The described strategy opens up unprecedented possibilities to build customized 3D cell microenvironments and biomimetic models, presenting various options toward the creation of binding sites for targeted cell immobilization onto 3D microscaffolds.

## 2 Results and Discussion

### 2.1 Site‐Specific Functionalization of 3D Microscaffolds

**Figure 1:**
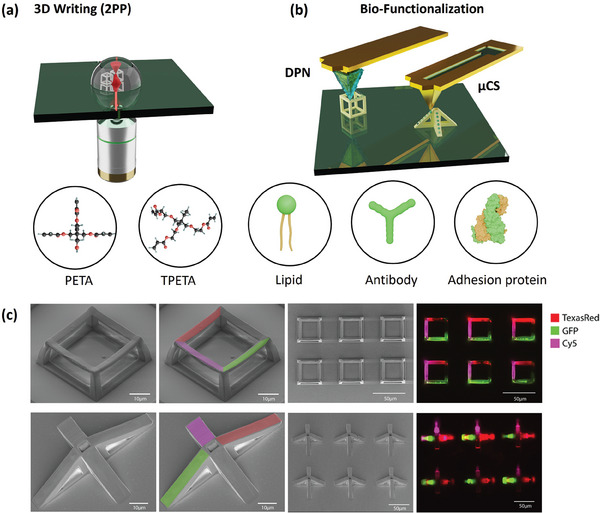
Schematic illustration of a) Two‐photon lithography process for 3D writing of microscaffolds using PETA and TPETA as monomers in the photoresist formulation and b) their biofunctionalization through DPN and/or µCS. c) SEM images of different microscaffolds for biofunctionalization, together with corresponding fluorescent images of the labelled phospholipid ink showing site‐specific functionalization.

We are presenting a novel approach for integrating diverse biomolecules onto 3D polymeric microscaffolds through SPL techniques. **Figure** **1**a,b schematically show the two main microfabrication steps that are proposed. First, a 3D microstructure is fabricated via 2PL, which is often employed to mimic the extracellular matrix (ECM). Two different model photoresists showing different young modulus, wettability and interaction with biomolecules have been used throughout the work: pentaerythritol triacrylate (PETA) and trimethylolpropane ethoxylate triacrylate (TPETA). The choice of these materials is grounded in their well‐established use^[^ ^29^ ^]^ and their distinct, well‐documented behaviors in protein adsorption. PETA is recognized for its protein‐adsorptive properties, making it suitable for applications where protein interaction is desired.

Conversely, TPETA is known for its protein‐repellent characteristics, making it ideal for scenarios where protein resistance is necessary. These contrasting properties of PETA and TPETA^[^ ^23^, ^30^ ^]^ provide a versatile platform for exploring various biomedical applications and optimizing the 3D cellular microenvironment. Detailed designs can be found in Section S1, Supporting Information (Figure S1, Supporting Information). The most relevant features are summarized in **Table** **1**. Biofunctionalization of the structures is subsequently conducted using atomic force microscopy (AFM)‐based direct‐write methods: DPN and/or µCS have been used depending on the ink properties and the desired patterning outcome. (DPN) is highly versatile, transferring various inks, including molecules and nanoparticles, to diverse substrates and enabling controlled assembly of nanomaterials. In DPN, a sharp tip is coated with molecular inks and positioned on a scanning probe microscope. The scanning probe setup precisely controls the vertical interaction of the tip with the substrate and guides the tip across the substrate. The ink on the tip is transferred to the substrate through a water meniscus, which forms from the moisture within the humidity chamber, linking the tip to the substrate. Advancements in DPN like parallel DPN (p‐DPN) and polymer pen lithography (PPL) have enhanced its applicability for high‐throughput and large‐scale patterning.^[^ ^26^, ^31^ ^]^ The detailed processesof 2PL and DPN are illustrated in schematics provided in Section S2, Supporting Information (Figure S2, Supporting Information).

**Table 1:** Monomers used for 2PL printing.

Photoresist	Name	Young's modulus	Wettability
PETA	pentaerythritol triacrylate	1 GPa^[^ ^33^ ^]^	Hydrophobic
TPETA	trimethylolpropane ethoxylate triacrylate	20 MPa^[^ ^30^ ^]^	Hydrophilic

This combination of techniques comprises relevant advantages regarding biofunctionalization in comparison to previously described approaches,^[^ ^23^, ^32^ ^]^ such as (i) variety of printable biomolecule inks that can be integrated onto 3D microstructures, (ii) the multiplexing capability and (iii) the achievable sub‐micrometer lateral resolution. The ability to employ various ink‐resist combinations demonstrates the versatility of our approach, which includes the deposition of both non‐water‐based and water‐based inks, on protein‐adsorptive (PETA) and protein‐repellent (TPETA) photoresists. This enables the integration onto 3D microstructures a wide range of biomolecules.

Another relevant aspect of our approach lies in the multiplexing capability, that is, the controlled integration of different inks on the same structure. This is exemplified in Figure 1c, where DPN patterning of a phospholipid with three distinct fluorophores was conducted. The fluorescence images showcase the precision and reliability of our method in achieving site‐specific functionalization with different biological compounds. Remarkably, the microstructures do not suffer any damage during patterning due to the minute forces applied with the probes. SEM images in Figure 1c capture the intact structure post DPN patterning, after printing on different types of surface geometries, from hanging flat surfaces to tilted structures. Additional examples can be found in the Section S3, Supporting Information.

Although the lateral resolution of the technique is dependent on the writing parameters^[^ ^34^ ^]^ (in particular relative humidity and dwell time) and it varies from one surface to another depending on their surface energy,^[^ ^35^ ^]^ our results demonstrate the capability to pattern on the used photoresist with at least sub‐1 µm lateral resolution (see Section S4, Supporting Information), underscoring the reliability of our approach in achieving finely detailed structures. Modifying the surface chemistry of the scaffold could further improve the lateral resolution of the biomolecule patterns, but it is less relevant in cell scaffolds, as cells display micron range sizes. For simplicity, most of the performed experiments shown subsequently were performed in square‐shaped printed structure, similar to the ones shown in Section S5, Supporting Information.

### 2.2 Surface Properties of Microscaffolds

**Figure 2:**
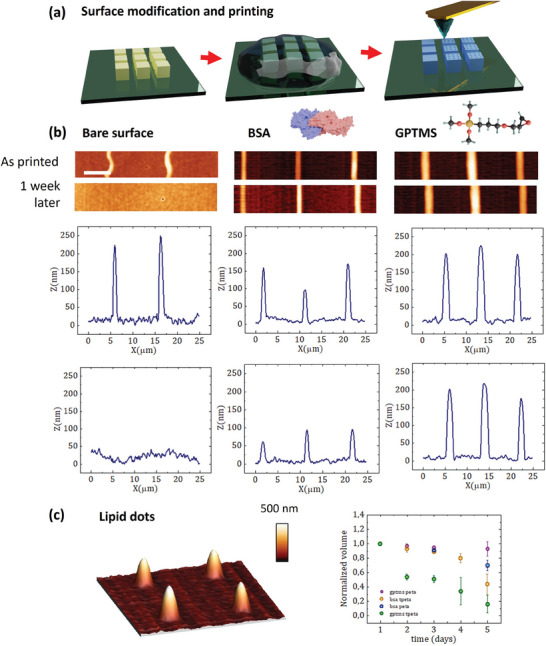
a) Schematic representation of the surface modification of 3D microscaffolds with BSA and GPTMS, followed by direct ink writing through DPN. b) AFM images of printed phospholipid lines on 3D microscaffolds (PETA) with different surface modifications, right after printing and 1 week after. c) 3D AFM image of printed phospholipid dots on GPTMS treated PETA. Graph illustrating the relative volume loss of phospholipid dots printed on PETA and TPETA photoresists with different surface modifications due to ink spreading over 5 days. Scale bar 5 µm.

The comprehension and control of surface properties of the microscaffolds is pivotal for effective functionalization through SPL printing approaches. In general, deposition of phospholipid patterns onto 3D microscaffolds presents two requirements. In the first place, hatching and slicing parameters need to be optimized to minimize surface roughness, which leads to undesired effects in direct ink writing, such as the formation of non‐continues lines (see Section S6, Supporting Information). Additionally, some inks, such as the phospholipid patterns tend to spread after some days in both of the used photoresist microscaffolds without any surface conditioning. Therefore, tuning the surface energy before direct ink writing becomes necessary. This a common procedure in 2D systems^[^ ^36^, ^37^ ^]^ and it can be extended to 2PL microscaffolds.^[^ ^14^ ^]^ To prevent lipid spreading, modifications of surface properties were conducted using two strategies validated in flat borosilicate substrates (see **Figure** **2**a). 40 µm × 40 µm × 10 µm sized microscaffolds of both PETA and TPETA photoresist were incubated with bovine serum albumin (BSA),^[^ ^38^ ^]^ a protein with 7 binding pockets to bind to fatty acids and (*3‐*glycidyloxypropyl)trimethoxysilane (GPTMS), a silanization process to produce an epoxy‐terminated substrate and increase its hydrophobicity.^[^ ^39^ ^]^ BSA and GPTMS are effectively used in various studies to create a biocompatible environment for biomolecules on the substrate.^[^ ^39^, ^40^, ^41^, ^42^, ^43^, ^44^, ^45^, ^46^ ^]^ Contact angle measurements (see Section S7, Supporting Information) reveal successful modification of the surfaces. The ink inhibition efficiency was evaluated on differently treated structures by printing test line patterns on microscaffolds using DPN. Subsequently, AFM and fluorescence microscopy were employed for inspection of the printed line and dot patterns of DOPC on microscaffolds. The shape and profiles of the printed lines were evaluated as printed and after 1 week. Representative outcomes are depicted in Figure 2b.

Comparisons between untreated structures and those modified with BSA and GPTMS reveal substantial differences in ink behavior. Untreated PETA and TPETA structures exhibit pronounced ink spreading, resulting in the eventual disappearance of the printed lipid line patterns within a few hours after printing. In contrast, structures treated with BSA and, more evidently, on GPTMS display stable line patterns, preserving the integrity of the printed lipid patterns for an extended period. Generally, L‐DPN written lipid structures will form during the writing process in stacks of lipid membranes with “tails‐out” configuration toward the air interface, then reconfigure into actual biomimetic “heads‐out” bilayers or stacks of bilayers.^[^ ^27^, ^47^, ^48^, ^49^, ^50^ ^]^ To probe the retainment of membrane fluidity (demonstrated for glass substrates)^[^ ^51^ ^]^ also in the case of the used polymeric materials, fluorescence recovery after photobleaching (FRAP) was conducted on substrates coated with BSA and GPTMS (Section S8, Figure S8, Supporting Information), showing that fluidity is preserved also in our case.

The influence of the two surface modification strategies was systematically investigated. Figure 2c provides a comparative assessment of the efficacy of surface modification using BSA and GPTMS on both PETA and TPETA microscaffolds. Over the course of a week, changes on printed patterns on four distinct photoresist and coating combinations were monitored: i) PETA treated with BSA, ii) PETA treated with GPTMS, iii) TPETA treated with BSA, and iv) TPETA treated with GPTMS. As a direct measure of lipid monolayers spreading over non flat surfaces endowed to many difficulties, the examination involved measuring the ink volume of the patterns and tracking their changes over 5 days. Nanodot geometries in Figure 2c were used as their volume can be easily quantified using the WSxM flooding tool.^[^ ^52^ ^]^ Results indicate that GPTMS treatment on PETA shows superior efficacy in retaining printed lipid structures, followed by BSA‐treated PETA, BSA‐treated TPETA, and, finally, GPTMS‐treated TPETA. While PETA possesses available OH groups that react to GPTMS forming covalent bonds, in the case of TPETA, GPTMS is just expected to be physiosorbed on its surface, which might reduce its efficacy when lipids are printed on top. The use of BSA to retain the lipid‐ink, instead, is a more biocompatible approach, with a lower performance as compared to GPTMS. Its stability on PETA is arguably higher than in TPETA, as this is a protein repellent photoresist.

The inhibition of lipid spreading on BSA and GPTMS‐coated PETA polymers compared to bare PETA surfaces can be attributed to specific interactions between the coatings and lipid molecules. (BSA) has a high affinity for binding lipids through its nonpolar amino acid side chains and cationic groups, creating strong hydrophobic and electrostatic interactions that anchor lipid molecules and prevent their free movement and spreading. This results in energetically favourable lipid accumulation on BSA‐coated surfaces, maintaining the stability of lipid structures. However, the exact mechanism behind the higher stability of lipid structures on GPTMS coated polymers requires further investigation. GPTMS presents glycidyloxypropyl groups with polar epoxy functional groups upon coating a surface. The enhanced stability of lipid structures may be attributed to the more favorable interaction between the polar lipid head groups and the polar surface. These interactions likely inhibit the free movement and spreading of the lipid molecules, resulting in reduced lipid spreading.^[^ ^36^, ^38^ ^]^ These finding emphasize the influence of surface modification in shaping the behavior of functional inks on 3D microscaffolds. The improved ink retention, facilitated by surface modification, presents opportunities to enhance the stability and durability of functional patterns on intricate microarchitectures.

Additional fluorescence imaging data are presented in the Section S9, Supporting Information, providing a comprehensive understanding of the impact of surface modification on lipid spreading across different photoresists.

### 2.3 Creation of Binding Sites for Cells by Integrating Different Biomolecules

Direct ink writing techniques, such as DPN and µCS have demonstrated great versatility in patterning diverse biomolecules, including DNA, proteins, peptides, lipids, and even biological entities as bacteria and viruses, either directly or indirectly.^[^ ^34^, ^53^, ^54^, ^55^ ^]^ Their gentle operating conditions and the possibility to control the exerted force are highly convenient for biofunctionalization, in comparison to other techniques that include harsh treatments like ultraviolet or electron‐beam irradiation. Achieving spatial control over the patterning of specific biomolecules is relevant to the creation of on‐demand microenvironments toward realistic in‐vitro models of cell‐environment interaction.^[^ ^56^ ^]^ Additionally, several tissue engineering applications, drug screening or cell–cell interaction studies require positioning of cells on 3D microscaffolds with a specific location. Toward this aim, in this part we are showing, not only capability to position biomolecules on 3D microscaffolds via SPL techniques with high spatial precision, but also opening different pathways to introduce binding sites for cell positioning, utilizing different strategies based on established conjugation.

**Figure 3:**
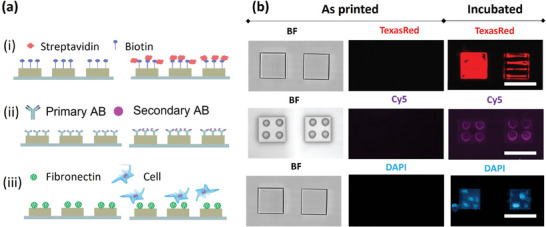
a) Schematic representations of i) biotinylated lipid printing on square structures via DPN followed by incubation with fluorescently labeled streptavidin, ii) AB printing via µCS, followed by incubation with fluorescently labeled secondary AB, and iii) µCS printing of fluorescently labeled fibronectin and as binding sites for cells. b) Optical microscopy images of structures with i) printed biotin bearing lipids as printed and after incubation with fluorescently labelled streptavidin, ii) printed antibodies before and after incubation with fluorescently labelled secondary antibodies, and iii) printed fluorescently labelled fibronectin before and after incubation with fibroblasts, labelled with DAPI.

The 3D microscaffold platforms were subjected to testing for three recognition assays, as depicted in **Figure** **3**, demonstrating distinct possible pathways to incorporate binding sites for spatial‐specific assembly of biomolecules or for cellular adhesion in 3D microscaffolds. These include biotin‐streptavidin binding, antigen/antibody (AB) binding and the incorporation of micro‐scale ECM protein arrays. **Table** **2** summarizes the different biomolecule inks that were written on squared‐shaped PETA microscaffold, together with the conjugation strategy.

**Table 2:** List of inks in or demonstration of printing on 3D microstructures.

Ink	Biomolecule (modification)	Solvent	Printing technique	Conjugation (modification)
BIOTINYL CAP PE	Phospholipid *(biotin)*	none	DPN	Streptavidin (TexasRed)
PRIMARY ANTI‐EPCAM ANTIBODY	AB *(none)*	Water/glycerol	µCS	Secondary AB (Cy5)
FIBRONECTIN	Protein *(GFP)*	Water/glycerol	µCS	Fibroblasts (DAPI)

First, we employed 3D microscaffolds of width 40 µm and height 10 µm for printing biotinylated lipids via DPN. Due to its favorable writing characteristics, DOPC serves as a carrier to enable the writing of materials that cannot be utilized in pure form for L‐DPN.^[^ ^57^ ^]^ Incorporating 5 mol% of 1,2‐dioleoyl‐sn‐glycero‐3‐phosphoethanolamine‐N‐(cap biotinyl) (Biotinyl CapPE) into this carrier produces a functional ink mixture. An AFM tip coated with biotinyl CapPE is applied to the target area on the PETA microscaffold, maneuvering over the surface to create the desired pattern. With its high affinity for binding streptavidin, biotin serves as a model for active sensor elements – a widely used concept in biotechnology for linking and immobilizing proteins and bioactive compounds. Binding experiments with fluorescently labeled streptavidin were conducted to explore the feasibility of using physisorbed biotinyl CapPE in liquid environments.

Figure 3a(i) depicts the schematic for streptavidin binding experiments. Two distinct Biotinyl‐CapPE patterns were printed on the microscaffolds: one with a uniform lipid layer covering the entire top surface (40 µm × 40 µm), and another with a few lines of 30 µm length with a separation of 10 µm. Following the blocking of non‐lipid‐functionalized areas with BSA in PBS to avoid non‐specific binding (see Section S10, Figure S9a, Supporting Information), the sample underwent incubation with a fluorescently labeled streptavidin solution. In the fluorescence image (Figure 3b(i)), the biotinyl CapPE patches illuminate, confirming the successful selective binding of streptavidin to the 3D microscaffold‐supported lipid membrane.

For water‐based inks, µCS emerged as the technique of choice, enabling printing of primary antibodies onto 3D microscaffolds (width 40 µm, height 10 µm). In µCS, a cantilever with a microchannel connected to an on‐chip ink reservoir contacts a substrate, allowing liquid transfer through capillary forces. As detailed in the methods section, we utilized the IgG category of immunoglobulins (Ig), the most prevalent type of antibodies in human serum for the experiments. Figure 3a(ii) illustrates the schematic for AB‐antigen binding experiments. Initially, the microscaffold platform was subjected to immobilization of the primary AB. Subsequently, the platform was incubated with the secondary AB acting as specific marker for the primary AB, labelled with Cy5. After incubation with the secondary AB (Figure 3b(ii)), the spots where the primary ABs were printed light up, confirming the successful selective binding of fluorescently labeled secondary antibodies.

The µCS technique was further employed to print a protein onto PETA microscaffolds. Fibronectin (FN), a commonly used glycoprotein to enhance the attachment of various cell types, under in‐vitro controlled conditions was chosen as an example of a printable protein present in the ECM and plasma. For subsequent visualization of the protein array, a fluorescently labeled FN (GFP labeled) was utilized for spotting, enabling convenient quality checks of pattern stability even after the removal of excess ink by washing. To prevent premature drying of the water‐based FN ink solution on the spotting tip, it was blended with 20 vol% glycerol. The ink‐filled Surface Patterning Tool (SPT) tip was brought into contact with the surface of a PETA microscaffold (width 40 µm, height 10 µm), allowing for the precise patterning of fibronectin spots on the microscaffold's surface as shown in Figure 3b(iii). Incubation with fibroblasts shows successful immobilization of cells onto the patterned FN spots.

The specific identification of the target (streptavidin/antigen/fibroblast) on microscaffolds functionalized with biotin/AB/FN validates the functionality of the immobilized functional moieties and demonstrates the suitability of the microscaffold platform for immunoassays.

### 2.4 Cell Immobilization Experiments

**Figure 4:**
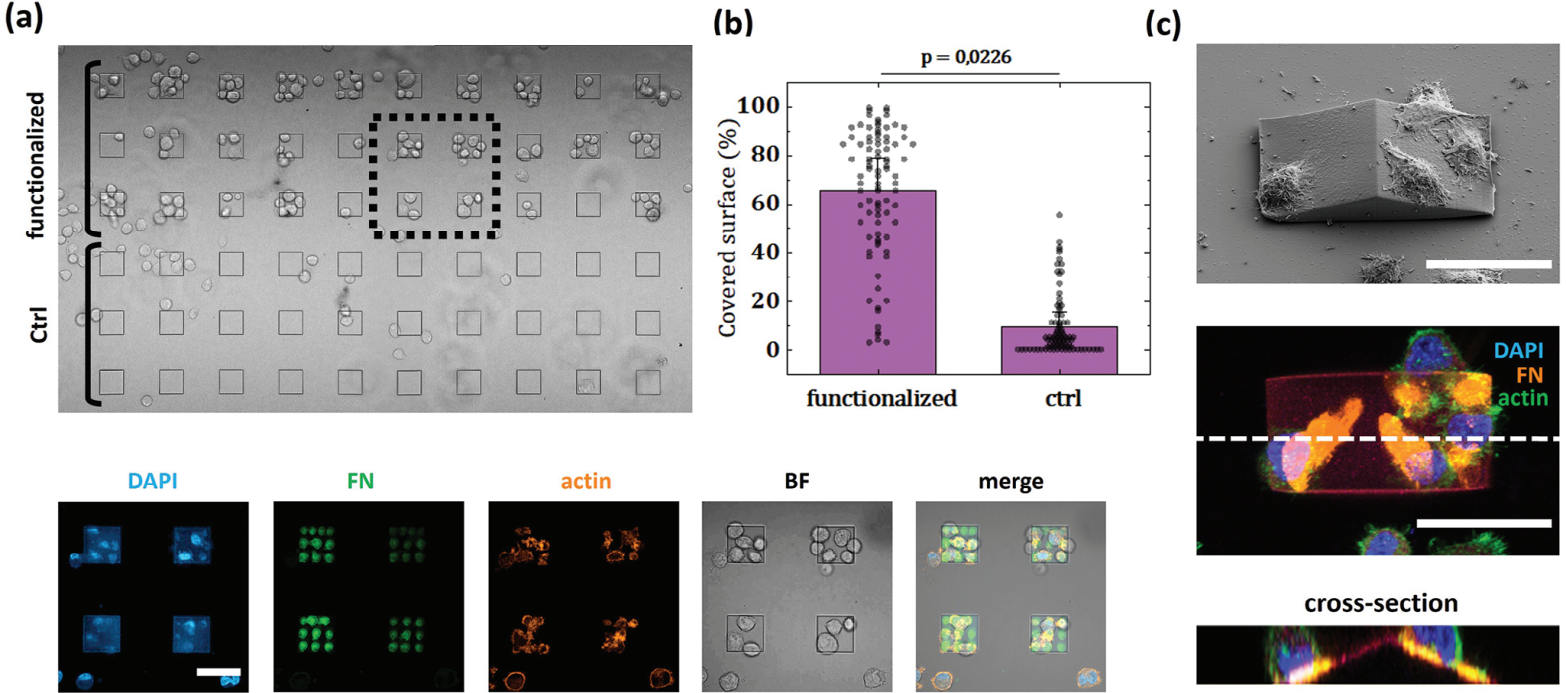
a) Balb/3T3 fibroblasts on PETA square structures, either functionalized with fibronectin printed via µCS, or non‐functionalized (ctrl). Fluorescence imaging of the dotted square area shows the FN pattern, as well as clear positioning of the cells on the functionalized structures. b) Area coverage by cells in the case of functionalization and non‐functionalization, respectively (N = 3 samples, n = 120 structures per condition). c) SEM imaging of PETA tilted structures and corresponding confocal immunofluorescence imaging (maximum intensity projection over 12 µm). The cross‐section corresponding to the dotted line confirms cell adhesion on tilted surfaces in correspondence to the functionalized areas. Scalebar 50 µm.

To systematically investigate the capability to induce cells adhesion, functionalized surfaces with fluorescently labeled FN via µCS were seeded with Balb/3T3 murine fibroblasts and incubated for 30 min. Although the formation of mature focal adhesions required longer incubation time, since vinculin immunostaining of cells did not show any significant protein clustering, as expected, cells clearly adhered preferentially on 2PL structures functionalized with FN, with respect to non‐functionalized surfaces (**Figure** **4**a). This behaviour could be quantified with statistical significance by measuring the portion of square structures occupied by adherent cells. Figure 4b shows the area covered by cells in the case of structures patterned with fibronectin and without functionalization, respectively (N = 3 samples, n = 120 structures per condition). Functionalized microstructures display 65% coverage in contrast to their non‐functionalized counterparts, showing 10% coverage due to non‐specific binding. Further experimental results are shown on Section S11, Supporting Information. The potential of µCS to modify 3D architectures was also demonstrated on pyramid‐like 2PL structures: as shown in Figure 4c, Balb/3T3 fibroblasts were able to adhere on tilted surfaces in correspondence to the areas where FN had been previously deposited.

## 3 Conclusion

In conclusion, we demonstrate a facile and efficient method for surface functionalization on 2PL‐printed microscaffolds. We report the possibility of realizing SPL‐based precise and spatially controlled biofunctionalization on 3D microscaffolds fabricated via 2PL for the first time. This method introduces the feasibility of expanding the functionalities for microscaffolds created from non‐functional photoresists by patterning big variety of biomolecule inks on the surface. Its compatibility with various photoresist‐ink combinations renders it a straightforward and versatile strategy for crafting functional 3D microscaffolds. In our study, we applied scanning probe lithographic techniques to two polymers, PETA and TPETA, which possess distinct mechanical properties and surface chemistries. This approach effectively demonstrated the versatility and robustness of our patterning methods across diverse materials. Our findings lay a solid foundation for future research to explore the impact of mechanical properties on patterning fidelity, specifically by investigating polymers with varying mechanical properties but identical surface chemistry.^[^ ^58^, ^59^, ^60^ ^]^ Understanding these dependencies will enhance the development and optimization of patterning techniques. The method's potential in the precise localization of cells on microscaffolds demonstrates its applicability for controlled cell positioning opening up additional biomedical applications, such as tissue engineering and cell therapy. We believe that the method is extensible to the patterning of non‐organic inks, such as nanoparticles or chemical functional groups to endow additional functionality to the microscaffolds by positioning active elements with high spatial control via SPL writing.

## 4 Experimental Section

### Chemicals and Materials

Standard solvents of p.a. grade (acetone, ethanol, isopropanol, choloroform, toluene) were obtained from Sigma‐Aldrich, Germany. DI water was produced by an Arium water purification system (Satorius, Germany). Pentaerythritol triacrylate (PETA) was obtained from Santa Cruz Biotechnology, Inc., Germany, trimethylolpropane ethoxylate triacrylate (TPETA) from Sigma‐Aldrich, USA and the photoinitiator Phenylbis (2,4,6‐trimethylbenzoyl) phosphine oxide (Irgacure 819) from Sigma‐Aldrich, USA. Bovine serum albumin (BSA), phosphate buffered saline (PBS), glycerol, and (3‐glycidyloxypropyl)trimethoxysilane (GPTMS) were obtained from Sigma‐Aldrich, Germany. Phospholipids 1,2‐dioleoyl‐sn‐glycero‐3‐phosphocholine (DOPC), 1,2‐dioleoyl‐sn‐glycero‐3‐phosphoethanolamine‐N‐(lissamine rhodamine B sulfonyl) (ammonium salt) (Liss Rhod PE), 1,2‐dioleoyl‐sn‐glycero‐3‐phosphoethanolamine‐N‐(Cyanine 5) (Cy5 PE), 1,2‐dioleoyl‐sn‐glycero‐3‐phosphoethanolamine‐N‐(7‐nitro‐2‐1,3‐benzoxadiazol‐4‐yl) (ammonium salt) (NBD PE), and 1,2‐dioleoyl‐sn‐glycero‐3‐phosphoethanolamine‐N‐(cap biotinyl) (sodium salt) (Biotinyl‐Cap‐PE) were all procured pre‐dissolved in chloroform from Avanti Polar Lipids, USA, and used as‐received. Anti‐EpCAM AB (Rabbit polyclonal, ab71916) and anti‐rabbit IgG Alexa Fluor 647 (donkey polyclonal, ab150075) were obtained from Abcam, UK. Fluorescently labeled fibronectin (HiLyte FluorTM 488 labeled) was obtained from Cytoskeleton, Inc., USA. Fluorescently labelled streptavidin (Streptavidin‐Cy3) was obtained from Thermo Fisher, Waltham, MA, USA.

### Photoresist Formulations

The photoresists were formulated by dissolving 20 mg of Irgacure 819 in 980 mg of PETA for PETA resist and in 980 mg of TPETA for TPETA resist. Each mixture was subjected to individual sonication for 120 min at 60 (C until the photoinitiator powder was fully dissolved.

### 3D Structure Fabrication via 2PL

Microscaffold structures were realized via a commercially available system (PPGT2 from Nanoscribe GmbH, Germany), equipped with a 780 nm femtosecond‐pulsed laser having a nominal power of 50 mW, along with a plan‐apochromat 63×/1.4 objective (Zeiss, Germany) and a transmittance of 76% (stated by the producer). The experimental procedure involved depositing a droplet of photoresist onto a 22 mm × 22 mm × 170 µm glass coverslip, which was meticulously cleaned through successive sonication in acetone, ethanol, and DI water, followed by drying under a nitrogen flow.

Subsequently, the prepared samples were secured in a suitable holder and exposed to laser radiation. The x‐y scanning was facilitated by the movement of galvanometric mirrors, while writing in the z‐direction was accomplished through the piezoelectric actuation of the sample holder. All structural designs were created using the software Blender and then imported into DeScribe (Nanoscribe GmbH, Germany). The scanning overlap parameters for flat structures were set at slicing 0.1 µm and hatching 0.1 µm, whereas for tilted structures, the parameters were slicing 0.02 µm and hatching 0.1 µm (see Figure S1, Supporting Information) The laser power and scan speed were set at 0.94 TW cm^−2^ and 8 mm s^−1^ for PETA monomer and 1.05 TW cm^−2^ and 8 mm s^−1^ for TPETA monomer. Irradiance was calculated from used laser powers according to Skliutas et al.^[^ ^61^ ^]^ Following the polymerization process, the samples underwent immersion in isopropanol for a minimum of 6 h, followed by drying with a nitrogen stream and subsequent storage in a light‐protected environment.

### Surface Modification of 2PL Structures

The glass substrates featuring 2PL fabricated 3D microscaffolds underwent surface modification either using BSA or GPTMS. For BSA coating, BSA was dissolved in PBS in a volumetric ratio of 10:90 and then incubated on the cleaned glass substrates with printed 3D microstructures for 1 h at room temperature (RT). Following the incubation period, the substrates were rinsed with DI water, dried under a nitrogen flow, and stored at RT until further use. For GPTMS coating, the substrates featuring 3D printed microstructures were immersed in a 2% v/v GPTMS in toluene solution for 1 h. The resulting silanized substrates were washed with acetone, ethanol and deionized water, dried using nitrogen, and stored at RT until subsequent use.

### Preparation of Functional Inks

In this study, three inks with distinct classes of biomaterials were employed, namely (I) phospholipid mixtures, (II) antibodies, and (III) fibronectin. (I) The inks for DPN with phospholipids (L‐DPN) were prepared by admixing the main carrier DOPC (20 mg ml^−1^) with appropriate quantities of fluorescently labeled or biotinylated phospholipids to achieve inks with 1 mol% concentration of Liss Rhod PE, Cy5 PE, or NBD PE, and/or 5 mol% Biotinyl‐CapPE. To enhance homogeneity, all mixtures underwent sonication for a brief duration. (II) For the primary AB ink, 0.5 µL of primary anti‐EpCAM AB (ab71916) was mixed with 50 µL of 10% BSA in PBS. (III) For the fibronectin ink, the fluorescently labeled fibronectin was dissolved in DI water to a final concentration of 2 mg mL^−1^ and mixed with 20% glycerol.

### Functionalization of 2PL Structures

Printing on the 3D microscaffolds was conducted using a Molecular Printer system (n.able GmbH, Germany), equipped with a humidity chamber and a light microscope. Two distinct types of printing probes were employed in this study: an A‐type cantilever probe (ACST, USA) for printing phospholipids via L‐DPN, and a Surface Patterning Tool (SPT‐S‐C30S, Bioforce Nanoscience) for printing fibronectin via µCS.

For L‐DPN, microfluidic inkwells (ACST, USA) were filled with 1 µL of the desired phospholipid inks, and the solvent (chloroform). After mounting of the probes in the Molecular Printer, ink loading was accomplished by immersing them in the channel of the inkwell for 10–15 min under high humidity conditions (80% RH). Following inking, any excess ink was removed by writing on a sacrificial area on the substrate, and a relative humidity of typically 30–40% and a speed of 20 µm·s^−1^ was maintained during the writing process.

For µCS, the SPT probes were cleaned and rendered hydrophilic by applying an oxygen plasma treatment (0.2 mbar, 100% O_2_, 100 W, 2 min, Atto Plasma cleaner, Diener electronic, Germany) prior to each printing experiment. Subsequent to cleaning, the probe's reservoir was loaded with 0.3–0.5 µL of the designated ink formulation and the probe was mounted in the Molecular printer. Then µCS spotting was performed with contacting time of ≈1 s.

### Streptavidin Binding Tests

To mitigate nonspecific binding, the samples functionalized with biotinylated phospholipids were initially subjected to a 30‐min incubation with a 10% BSA solution at RT. Subsequently, the demonstration of biofunctionalization was done by incubating the samples with a 50 µL solution of a mixture of 1 µl streptavidin‐Cy3 (1 mg·mL^−1^) in 200 µl PBS for an additional 30 min. Before fluorescence imaging, the samples were washed by pipetting on and off 50 µL of PBS thrice and once with 50 µL of DI water.

### Immunostaining

Following the printing of the primary AB onto the 3D microstructures, an incubation step ensued with 0.5 µL of secondary anti‐rabbit IgG Alexa Fluor 647 (ab150075) in 50 µL of a 10% BSA in PBS solution, lasting for 1 h. Subsequently, the samples underwent three washes by immersion in 100 mL of deionized (DI) water, after which they were imaged using a fluorescence microscope.

### Cell Experiments and Immunofluorescence

For cell adhesion experiments, FN was printed via µCS on either square or tilted 2PL microscaffolds. Prior to cell seeding, the edges and the lower surface of the coverslip were carefully wiped with ethanol to minimize the risk of contamination from previous treatments. Balb/3T3 mouse fibroblasts were cultured in a T75 flask in DMEM supplemented with 10% foetal bovine serum (FBS), 2 mM L‐glutamine, 100 IU mL^−1^ penicillin, and 100 µg mL^−1^ streptomycin, at 37 °C and 5% CO_2_. Cells were trypsinized, pelleted and resuspended in 1 mL of FBS‐free DMEM supplemented with 2 mM L‐glutamine, 100 IU mL^−1^ penicillin and 100 µg mL^−1^ streptomycin, and seeded on the structures. After 30 min, samples were gently washed several times with fresh culture medium to remove cells which did not adhere on the FN spots. Fixation with paraformaldehyde 4% (in PBS) for 15 min followed. For immunofluorescence, samples were first washed three times with PBS containing 0.1% Triton X‐100 (PBST) for 5 min, and incubated for 1 h with a monoclonal mouse primary anti‐vinculin antibody (sc‐73614 from Santa Cruz Biotechnology, 0.4 µg mL^−1^) in PBS+1% bovine serum albumin (BSA). After incubation, samples were washed with PBST for three times and incubated for 1 h with a goat anti‐mouse AlexaFluor647‐coupled secondary AB (ThermoFisher, 2 µg mL^−1^). Actin filaments were stained with ActinGreen488 stain or ActinRed555 stain (from Tebubio, Cat. No. C052 and C053, respectively), diluted 1:500 and 1:800 in PBS+1% BSA, respectively. In both cases, the starting stock solution was obtained according to the recommendations of the Producer, that is, by dissolving the whole amount of actin probes in 1.5 mL of methanol. Nuclei were stained with DAPI (1 µg mL^−1^). After a final rinse with PBS, samples were analysed via confocal microscopy.

### Optical Imaging

Microscope images of the printed patterns were captured using a Eclipse 80i fluorescence microscope (Nikon, Germany) featuring Intensilight illumination (Nikon, Japan). Imaging was facilitated by a DSQi2 camera (Nikon, Germany), employing TexasRed filters (excitation/emission wavelength: 559/630 nm, color‐coded red), FITC filters (475/530 nm, color‐coded green), and Cy5 filters (604/712 nm, color‐coded purple). For cells experiments, a Nikon A1R+ confocal microscope, using excitation wavelengths of 405, 488, 561, and 647 nm, and emission ranges of 425–475, 500–550, 575–625, and 650–700 nm, respectively. Quantification of the portion of the surface of 2PL microscaffolds occupied by Balb/3T3 cells was carried out according to a protocol described elsewhere.

### Fluorescence Recovery After Photobleaching Mobility Study

FRAP experiments were conducted using an Eclipse 80i fluorescence microscope (Nikon, Germany) equipped with Intensilight illumination (Nikon, Japan). Imaging was performed with a DSQi2 camera (Nikon, Germany) and TexasRed filters (excitation/emission wavelength: 559/630 nm, color‐coded red). The bleaching spot was ≈80 µm in diameter. Bleaching was carried out using Bright Field (white light) at maximum intensity for 20 min. The recovery process was documented from 0 to 40 min, with recordings taken at 1‐min intervals from 0 to 5 min, and then at 10‐min intervals from 10 to 40 min to track fluorescence intensity. Fluorescence recovery was quantified by averaging the intensity within a fixed circular region.

The diffusion coefficients were determined by fitting the obtained normalized intensity curve to the following function:

(1) $$I_{\mathrm{norm}}\;\left(t\right)\;=\;1-\frac{r_{0}}{w}\;\exp\;\left(-\frac{4\mathrm{Dt}}{w^{2}}\right)$$

where I_norm_ is the normalized intensity, r_0_ is the radius of the bleached spot, w is the width of the Gaussian profile of the fluorescent light and D is the diffusion coefficient.

### Atomic Force Microscopy Imaging

The AFM characterization of the printed patterns on 3D microscaffolds was conducted using a Dimension Icon system (Bruker, Germany) in tapping mode. Tap300AI‐G probes (Budget sensors, Bulgaria) featuring a resonant frequency of 300 kHz and a spring constant of 40 N m^−1^ were employed for the analysis. The obtained images were processed and analyzed using WSxM software.

### Scanning Electron Microscopy Imaging

The imaging of the printed 3D microstructures was carried out using SEM LEO‐1530 (Zeiss, Germany). Samples were sputtered with 10 nm of gold (Cressington Sputter Coater 108auto, Germany) prior to SEM imaging. The acceleration voltage and working distance were set at 5 kV and 9.2 mm, respectively. For cell imaging, cells previously inspected via confocal microscopy were dehydrated by immersing samples in a series of ethanol solutions of increasing concentrations (50%, 70%, 80%, 95%, 100% twice) for 10 min each. Afterward, samples were washed in pure hexamethyldisilazane (HMDS) for 10 min twice. After evaporation, samples were sputtered with 4 nm of gold (Baltec SCD500) and inspected with a Zeiss Sigma 300 VP (acceleration voltage 3 kV).

### Statistical Analysis

Statistical analysis of the cell immobilization experiments in Figure 4 was carried out by using 3 different samples with 120 structures per condition (N = 3, n = 120). Differences were regarded as statistically significant at *p* < 0.0226. All data statistics and charts were obtained by using Origin 2021.

## Conflict of Interest

The authors declare no conflict of interest.

## Supporting information



Supporting Information

## Data Availability

The data that support the findings of this study are available from the corresponding author upon reasonable request.
