# Supplementary material for: Site‐Selective Biofunctionalization of 3D Microstructures Via Direct Ink Writing
Source: Small. 2024 Sep 18;20(51):2404429. doi: 10.1002/smll.202404429 (PMC11657036; doi:10.1002/smll.202404429)
Supplement: Supplementary file 1 — Supporting Information [file SMLL-20-2404429-s001.pdf]

NANO · MICRO
small

Supporting Information

for *Small*, DOI 10.1002/smll.202404429

Site-Selective Biofunctionalization of 3D Microstructures Via Direct Ink Writing

George Mathew, Enrico Domenico Lemma, Dalila Fontana, Chunting Zhong, Alberto Rainer, Sylwia Sekula-Neuner, Jasmin Aghassi-Hagmann, Michael Hirtz and Eider Berganza**

Supporting Information

Site-Selective Biofunctionalization of 3D Microstructures Via Direct Ink Writing

George Mathew, Enrico Domenico Lemma, Dalila Fontana, Alberto Rainer, Sylwia Sekula-Neuner, Jasmin Aghassi-Hagmann, Michael Hirtz, and Eider Berganza**

Supporting Information 1: Schematic representation of structural designs employed in fabricating the micro-scaffolds using 2PL presented in this study.

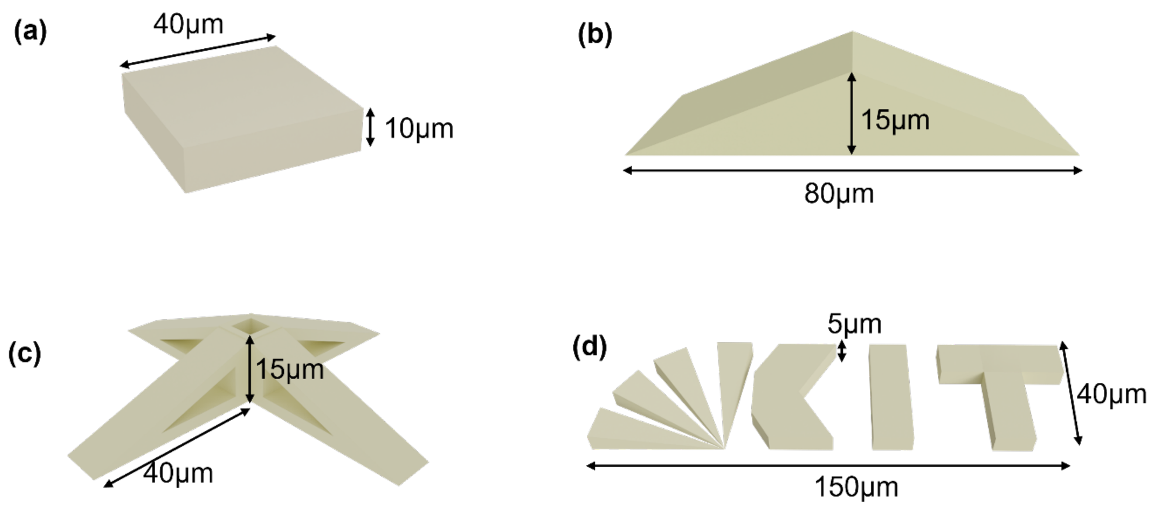

Figure S1. (a) 3D geometrical design with planar top surface: This micro-scaffold design served as a substrate for printing various biomolecules and is utilized in experiments to investigate micro-scaffold surface properties (slicing 0.1 µm, hatching 0.1 µm), (b) 3D geometrical design with inclined top surfaces: This design served as a substrate for cell immobilization experiments (slicing 0.02 µm, hatching 0.1 µm), (c) design of another inclined micro-scaffold utilized as a substrate for multiplexing experiment (slicing 0.02 µm, hatching 0.1 µm) and (d) a representation of Karlsruhe Institute of Technology (KIT) logo fabricated using 2PL (slicing 0.1 µm, hatching 0.1 µm)

Supporting Information 2: Detailed Schematics of 2PL and DPN Processes

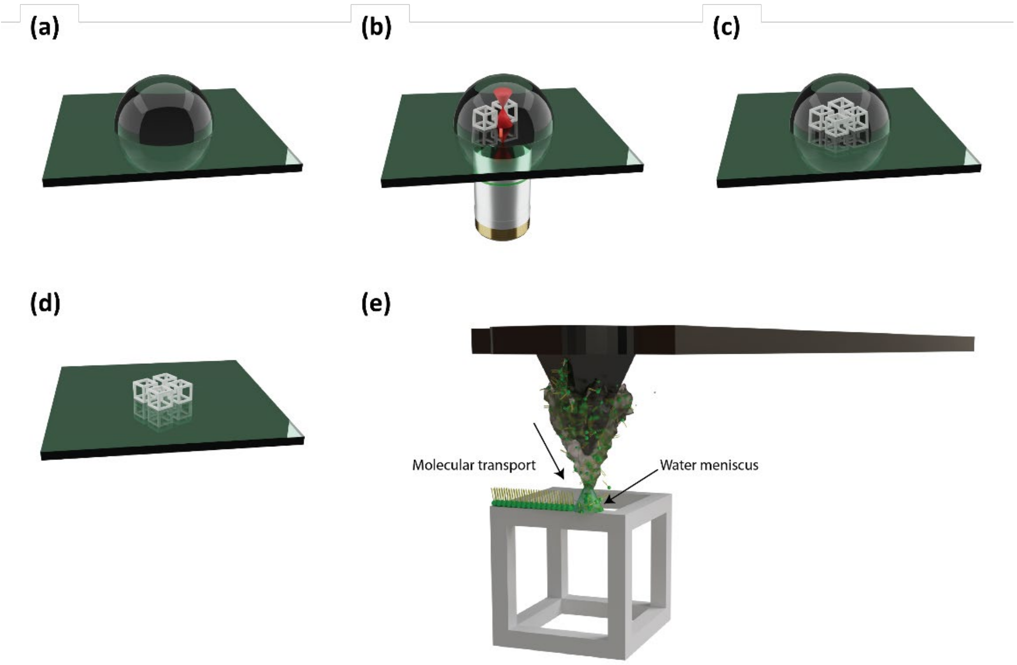

Figure S2. (a) A liquid photoresist is applied onto a glass coverslip. (b) Focusing a femtosecond pulsed laser into the liquid resist results in two-photon absorption occurring exclusively within the focal volume, inducing spatially controlled cross-linking. (c) By maneuvering the laser focus throughout the photoresist, arbitrary 3D microstructures can be fabricated. (d) The substrate is then immersed in a solvent and rinsed to remove any non-cross-linked liquid resist. (e) A plasma-treated tip loaded with ink forms a water meniscus upon contact with the polymer surface, facilitating the transport of lipids or molecules to the polymer surface.

Supporting Information 3: Further biofunctionalization examples.

The KIT logos, produced through 2PL, undergo functionalization processes involving Fibronectin attachment via µCS and lipid incorporation via DPN. Phospholipids are fluorescently labeled with GFP and Cy5, whereas Fibronectin is labeled with rhodamine. The KIT logo comprises both flat and tilted structures.

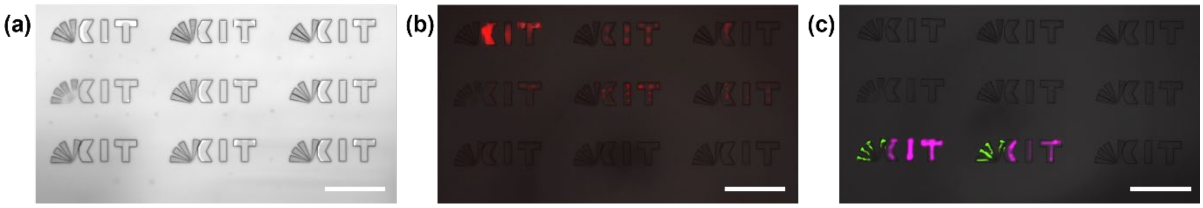

Figure S3. (a) Bright field image of KIT logo combining flat (letters) and tilted structures (sketch). Fluorescent images of their functionalization (b) Fibronectin spots printed with µCS and (c) Lipids printed with DPN. Scalebar 100 µm.

Supporting Information 4: Lateral resolution

Figure S4 depicts the printing of lipid lines and spots on untreated and GPTMS-treated PETA micro-scaffolds, showcasing the impact of writing speed and dwell time on the dimensions of the printed features. Figure S4a illustrates lipid lines printed on untreated PETA micro-scaffolds at a relatively high writing speed, i.e., 20 µm/s.

Figure S4(b) exhibits the lipid lines printed on GPTMS-treated PETA micro-scaffolds, demonstrating variations in line dimensions with different writing speeds. Table S1 provides a comprehensive overview of how different writing speeds influence the height and width (FWHM) of the printed lines, showing the trend reported in previous articles related to lipid-DPN.[38] Figure S4(c) displays lipid spots on GPTMS-treated PETA micro-scaffolds with a periodicity of 10 µm and a dwell time of 10 ms.

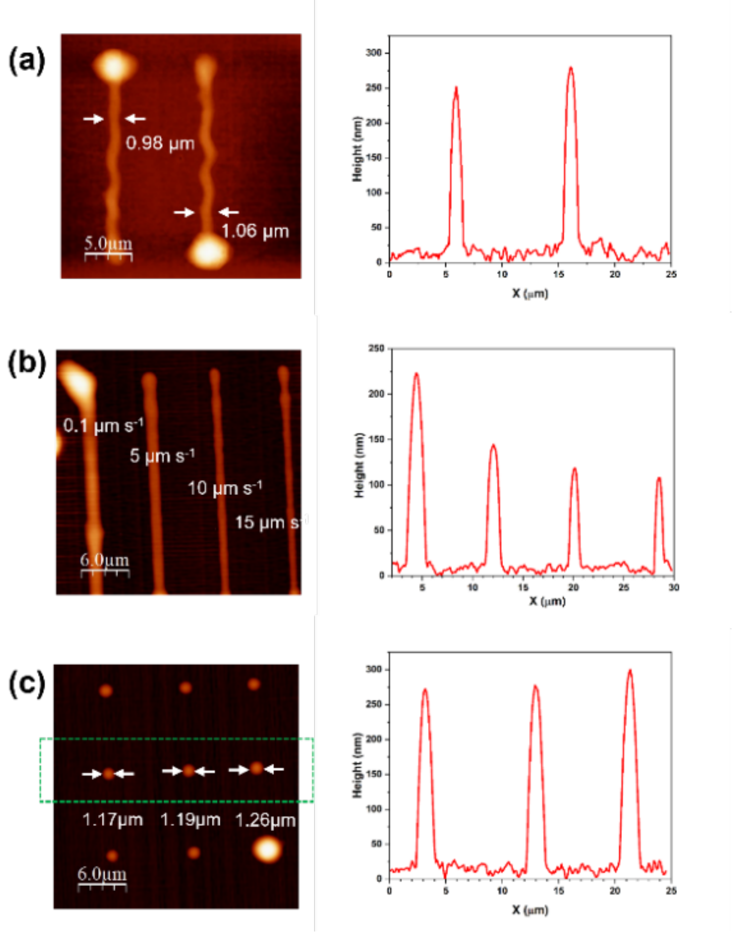

Figure S4. (a) Phospholipid lines written on bare PETA surfaces at 20 µm/s. (b) Phospholipid lines written on modified PETA surfaces with GPTMS at increasing speeds. (c) Dots patterned on GPTMS/PETA surfaces with 10 ms of dwell time.

Table S1. Height and FWHM of lipid lines obtained on GPTMS coated PETA micro-scaffolds.

Writing speed (µm s-1)	Height of line (nm)	Width of line (FWHM) (µm)
0.1	222.76	1.37
5	144.24	1.17
10	118.42	0.90
15	107.88	0.77

Supporting Information 5: 3D printed microsquares

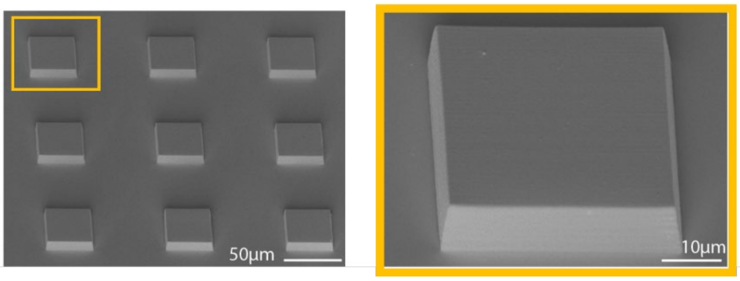

Figure S5. Scanning Electron Microscope image of microcubes used for some of the proof-of-principle experiments.

Supporting Information 6: Considerations on surface roughness

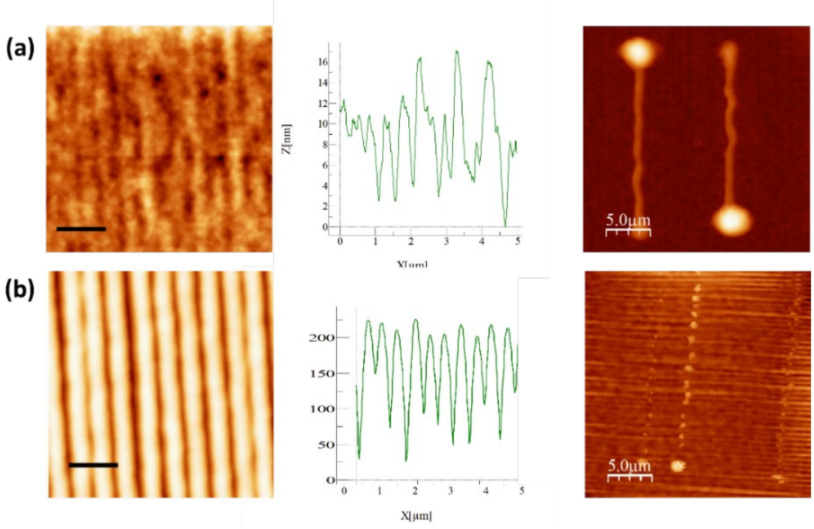

Figure S6. AFM image and profiles of the surface of PETA microstructures 3D printed with hatching distances (a) 100 nm and (b) 300 nm. DOPC lines written on microstructures with different surface roughness show different outcomes.

Supporting Information 7: Surface characterization of modified PETA and TPETA micro-scaffolds

Table S2. Contact angle values on different surfaces.

	bare	GPTMS	BSA
**Borosilicate (glass)**	28.15	47.75	53.20
**PETA**	69.40	52.35	62.05
**TPETA**	45.50	54.40	44.95

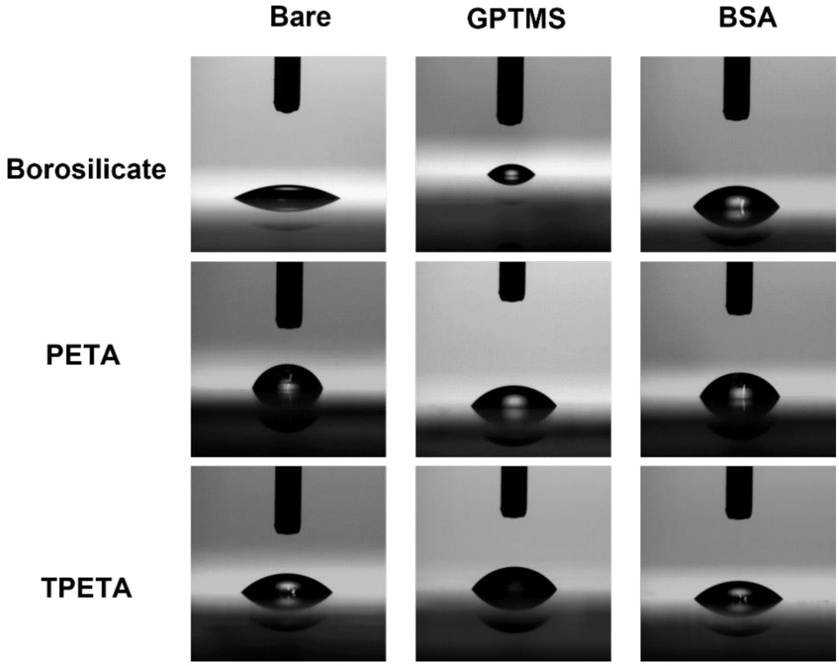

Figure S7. Contact angle measurements on borosilicate (control), PETA and TPETA surfaces with no modification, GPTMS and BSA coatings.

Supporting Information 8: Fluorescent Recovery After Photobleaching (FRAP)

FRAP experiments are conducted onto lipid patches patterned with DPN on the different studied surfaces: PETA, BSA/PETA, GPTMS/PETA, TPETA, BSA/TPETA, GPTMS/TPETA. The recovery of the bleaching is tracked taking fluorescent images at different times, as shown in Figure S8. The normalized intensity data is fitted to an exponentially decreasing function given by

$$I_{norm}\,(t) = 1 - \frac{r_0}{w}\exp\left(-\frac{4Dt}{w^2}\right)$$

where I_{norm} is the normalized intensity, r_0 is the radius of the bleached spot, w is the width of the Gaussian profile of the fluorescent light and D is the diffusion coefficient.

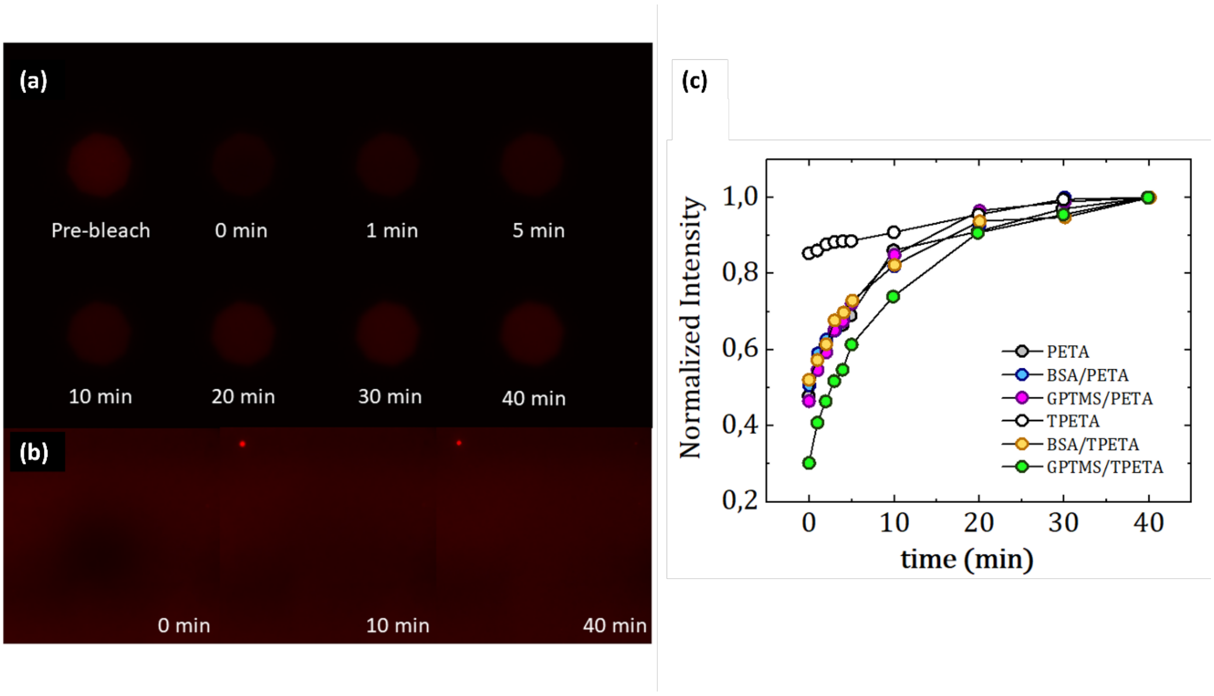

Figure S8. FRAP experiment on DOPC Rhod-PE printed on various functionalized substrates. (a) Time-lapse imaging of fluorescence recovery after photobleaching on DOPC Rhod-PE printed on BSA-coated PETA polymer, with the aperture partially closed, exposing only a small circular area to high-intensity light, resulting in photobleaching, (b) The same field of view is shown with the aperture completely open, revealing the photobleached dark circular region alongside the surrounding non-bleached areas, and (c) representative FRAP curves, normalized to pre-bleach intensities, for BSA and GPTMS-coated PETA and TPETA polymers.

The diffusion coefficients obtained on different surfaces are gathered in Table S3. Overall, diffusion coefficient for lipids in cell membranes typically lies within the 1-10 μm²/s range. The obtained values fit well into this range.

Table S3. Calculated diffusion coefficients for printed lipids on different surfaces

**Surface**	**D (μm²/s)**
PETA	2.85
PETA/BSA	2.65
PETA/GPTMS	2.98
TPETA	-
TPETA/BSA	1.93
TPETA/GPTMS	1.65

Supporting Information 9: Lipid ink spreading on modified surfaces

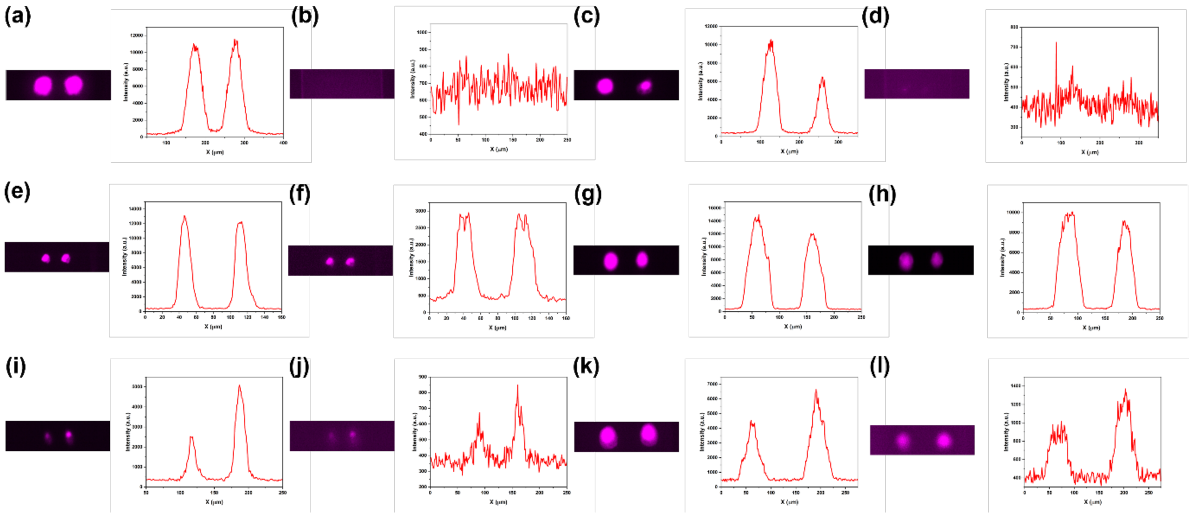

Figure S9. Comparison of Fluorescence Spectra for Lipid Ink Spreading on Unmodified and Modified Surfaces of PETA and TPETA Micro-scaffolds: (a) Unmodified PETA on Day 1, (b) Unmodified PETA on Day 4, (c) Unmodified TPETA on Day 1, (d) Unmodified TPETA on Day 4, (e) BSA Modified PETA on Day 1, (f) BSA Modified PETA on Day 4, (g) GPTMS Modified PETA on Day 1, (h) GPTMS Modified PETA on Day 4, (i) BSA Modified TPETA on Day 1, (j) BSA Modified TPETA on Day 4, (k) GPTMS Modified TPETA on Day 1, (l) GPTMS Modified TPETA on Day 4.

Systematic experiments were conducted using AFM imaging to determine the average volume of lipid ink deposited on both unmodified and modified PETA and TPETA micro-scaffolds, aiming to quantify differences in spreading behavior. Detailed information regarding the volume calculation methodology is provided elsewhere.[36]

Supporting Information 10: BSA utilization in blocking and surface modification

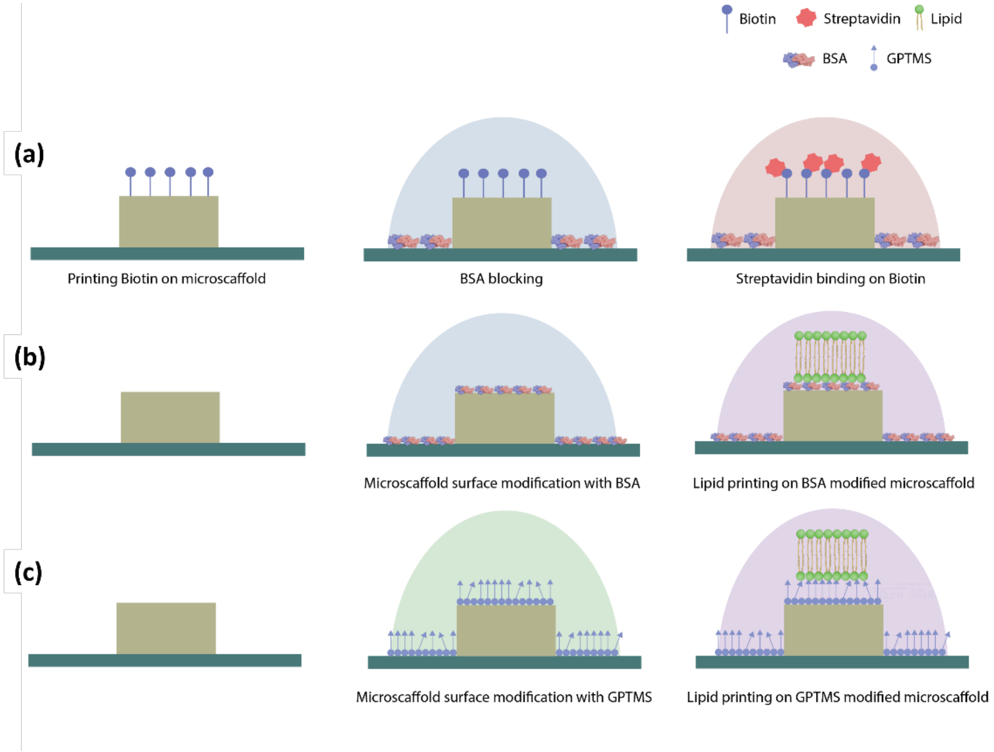

Figure S10. (a) BSA utilized as a blocking agent on biotin-coated microstructures prior to streptavidin binding, (b) BSA employed for surface modification of microstructures before lipid printing, and (c) GPTMS used for surface modification of microstructures before lipid printing.

Supporting Information 11: Cell binding on fibronectin coated micro-scaffolds

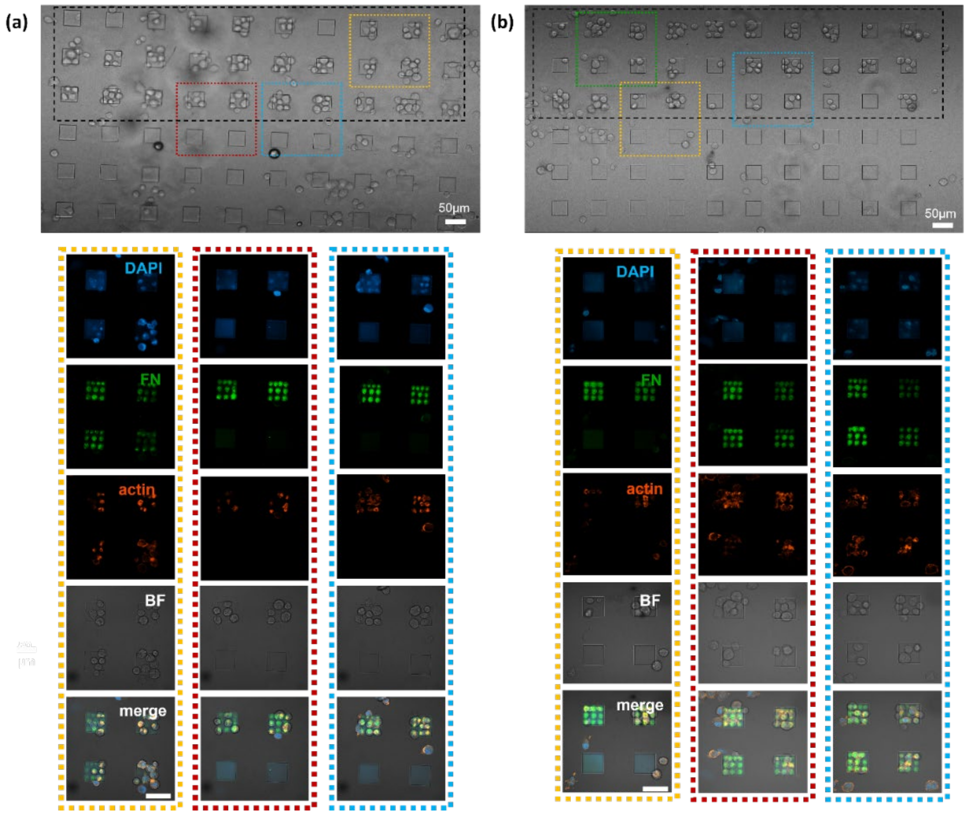

Figure S11. Supplementary bright field and fluorescent images depict two samples featuring micro-scaffolds functionalized with fibronectin for enhanced cell binding. In images (a) and (b), the micro-scaffolds within the black dotted rectangular regions are functionalized with fibronectin (FN), while those outside remain unmodified.
